# Profiling of 2-Acetyl-1-Pyrroline and Other Volatile Compounds in Raw and Cooked Rice of Traditional and Improved Varieties of India

**DOI:** 10.3390/foods10081917

**Published:** 2021-08-18

**Authors:** Deepak Kasote, Vivek Kumar Singh, Haritha Bollinedi, Ashok Kumar Singh, Nese Sreenivasulu, Ahmed Regina

**Affiliations:** 1 Centre of Excellence in Rice Value Addition (CERVA), International Rice Research Institute (IRRI)—South Asia Regional Centre (ISARC), Varanasi 221106, India; d.kasote@irri.org (D.K.); vivek.singh@irri.org (V.K.S.); a.regina@irri.org (A.R.); 2 Division of Genetics, ICAR—Indian Agricultural Research Institute (IARI), New Delhi 110012, India; haritha.agrico@gmail.com (H.B.); aks_gene@yahoo.com (A.K.S.); 3 Consumer-Driven Grain Quality and Nutrition Research Unit, International Rice Research Institute, Los Baños 4031, Philippines

**Keywords:** aroma, 2-acetyl-1-pyrroline, volatiles, rice, cooked, HS-SPME-GC-MS, traditional landraces, metabolomics

## Abstract

Herein, optimized headspace solid phase microextraction with gas chromatography–tandem mass spectrometry (HS-SPME-GC-MS/MS) was used to estimate the 2-acetyl-1-pyrroline (2-AP) in raw and cooked rice samples of ten different traditional and improved varieties. Furthermore, HS-SPME-GC-MS-based volatile profiling was subjected to untargeted analyses to identify major odorants in raw and cooked rice samples, and to understand chemical proximities among volatile profiles. Results showed that 2-AP content was remarkably increased in cooked rice compared to raw. Among the varieties studied, Pusa-1652 (Improved Kala Namak) and Kala Namak-2 were superior in the 2-AP content than Basmati varieties. Additionally, Govind Bhog, Kala Jeera and Jeera-32 had 2-AP content equivalent to or superior to Basmati rice varieties. Altogether, 18 and 22 volatiles were identified in the raw and cooked rice samples studied, respectively. Of these, ethyl butyrate, ethyl 3-methylbutanoate, 2-undecanone, ethyl benzoate, ethyl benzeneacetate, 2-methylnaphthalene, and 1-methylnaphthalene were characteristically detected in the cooked rice. The high amount of 2-ethyl-1-hexanol was uniquely found in raw rice samples, which can be a marker compound for freshly milled rice. Along with 2-AP, butanoic acid and benzoic acid derivatives, phenylethyl alcohol, ethyl 3-hydroxybutyrate, and indole may be responsible for the overall perceived characteristic Basmati-like aroma in cooked rice.

## 1. Introduction

At present, over 150 different volatile compounds have been reported in raw and cooked milled rice, which are mainly from the classes, alkanes, aldehydes, alcohols, esters, ketones, phenols, fatty acids, benzyl derivatives, enones, furans, furanones, monoterpenoids, sesquiterpenoids, naphthalenes, xylenes, pyridines, and pyrroles [1,2,3,4]. However, only some of these volatiles are responsible for the overall perceived aroma of rice [5]. Among these, the 2-acetyl-1-pyrroline (2-AP), aldehydes, heterocyclics, and alcohols are the primary odorants [6].

In cooked rice, the perceived aroma depends not only on the levels of primary odorant but also on their interactions [7]. For the past several decades, 2-AP has been considered a primary determinant of flavor in aromatic rice due to the loss of function of betaine aldehyde dehydrogenase 2 (*OsBADH2*) and it has frequently been used as a marker compound to distinguish aromatic and non-aromatic rice varieties [8]. However, marker association of *OsBADH2* is not tightly linked to the quantitative variation of 2-AP. Hence, the level of 2-AP in rice samples is usually assessed to predict aroma intensity. The genetic differences are primarily responsible for the variation in 2-AP contents. Besides this, the content of 2-AP is also found to be influenced by storage conditions, harvesting, and evaluation methods [6,9]. In rice, 2-AP is enzymatically biosynthesized during the plant growth and may also be formed at the time of heating and cooking operations as part of the Maillard reaction [10]. However, the formation or release of 2-AP during cooking is not well understood, due to various reasons including the lack of a well-optimized analytical method [6].

In the present study, multiple reaction monitoring (MRM) mode headspace solid phase microextraction with gas chromatography–tandem mass spectrometry (HS-SPME-GC-MS/MS) was optimized to estimate 2-AP contents in raw and cooked rice samples of traditional and improved rice varieties. Moreover, HS-SPME-GC-MS-based metabolite analysis was conducted to identify other critical odorants in raw and cooked rice samples of these varieties through the untargeted metabolomics approach. The interaction of volatiles and the proximities between volatile profiles were also investigated by computing Pearson’s correlation. 

## 2. Materials and Methods

### 2.1. Rice Samples

Grain samples of ten rice varieties, comprising four improved varieties (IR-64, Pusa Basmati-1, Pusa Basmati-1509, and Pusa 1652 (Improved Kala Namak)) and six landraces (Jeera 32, Govind Bhog, Kala Jeera, Kala Nuniya, Kala Namak-1, and Kala Namak-2) were obtained from the Farm Seed Storage Facility of the International Rice Research Institute, South Asia Regional Centre (ISARC) (Figure S1). Based on aroma testing through the sensory approach, Basmati-1, Pusa Basmati-1509, Pusa 1652 (Improved Kala Namak), Kala Namak-2, Jeera 32, Kala Jeera, and Govind Bhog were confirmed as aromatic germplasm (data not shown). Although Kala Nuniya and Kalanamak-1 are considered as aromatic rice varieties, our sensory test revealed only mild-aromatic rice of these samples. All varieties were cultivated in the wet season for two consecutive years (2019 and 2020) in the ISARC farm, Varanasi, India (25.282° N latitude, 82.956° E longitude). The plot size was 1 m × 5 m with 20 cm row spacing. The average accumulated precipitations in 2019 and 2020 were 1114.7 mm and 1164.5 mm, respectively. Initially, seeds were sown in a nursery in the last week of June, and twenty-one days old seedlings were transplanted manually in the main field. After maturity, the paddy was harvested and sun-dried until moisture content was reduced to about 13%. Paddy samples of all the above varieties were stored in cold conditions until further processing. All paddy samples were de-hulled and milled with a rice-dehulling machine (PAZ-1/DTA, Testing Rice Mill, Zaccaria S/A, Brazil) to obtain milled rice.

### 2.2. Chemicals

The authentic standard 2-AP (10 mg, ~10% *w/w* in toluene) was procured from Clearsynth, India. The C7–C40 saturated alkane mixture (1000 mgL^−1^ each component in hexane) was obtained from Supelco, Merck, India. The internal standard 2,6-dimethylpyridine (2,6-DMP), indole, naphthalene, and 1-hexanol were purchased from Sigma-Aldrich, India. All other chemicals used in this study were of analytical grade.

### 2.3. Optimization of HS-SPME Conditions

The extraction efficiency of three coated fibers such as 85 µm carboxen/polydimethylsiloxane (CAR/PDMS), 65 µm polydimethylsiloxane/divinylbenzene (PDMS/DVB), and 50/30 µm divinylbenzene/carboxen/polydimethylsiloxane (DVB/CAR/PDMS) were tested before the implementation of the experiment. For this study, a 1.0 g rice grain sample was placed in a 20 mL headspace vial, and extraction was carried out at 90 °C for 30 min. The optimal condition for the extraction of 2-AP from raw rice grain was tested at different sample weights (0.50, 1.00, and 1.50 g) using incubation/extraction temperatures of 30, 60, and 90 °C and extraction times of 5, 15, and 30 min. The amount of water (0.25, 0.50, and 1.00 mL) required to extract the optimal amount of 2-AP from cooked rice (1.0 g in 20 mL headspace vial) was determined at the optimized extraction time and temperature (15 min at 60 °C).

### 2.4. HS-SPME Based Extraction of Volatiles from Raw and Cooked Rice

One gram of rice grain sample was weighed in the 20 mL headspace vial, and then 5 µL (20 µg mL^−1^) of 2,6-DMP was added to it as an internal standard. For the extraction of volatiles from cooked rice, an aliquot of 0.25 mL of ultra-pure water was added to the 20 mL headspace vial containing the 1.0 g rice grain sample before the addition of an internal standard. The sample vial was then sealed with a PTFE/silicon cap, and shifted on an autosampler (PAL RSI 120, CTC Analytics, Zwingen, Switzerland) equipped with a gas chromatography system (7890B, Agilent Technologies, Shanghai, China) and triple quad mass spectrometer (7000B, Agilent Technologies, Santa Clara, CA, USA). A 50/30 µm DVB/CAR/PDMS fiber needle (57298U, Supelco, Bellefonte, PA, USA) was used to absorb volatile compounds, including 2-AP, in the headspace vial. The fiber was pre-conditioned for 10 min at 270 °C, and the extraction was carried out at the incubation temperature of 60 °C for 15 min. Then, the sample was injected in the inlet port (set temperature was 250 °C) and processed in split mode (5:1). The sample desorption time was 0.5 min. Finally, the fiber was post-conditioned for 30 min at 270 °C before the following analysis.

### 2.5. Determination of 2-AP Using HS-SPME-GC-MS/MS

For the estimation of 2-AP, DB-WAX column (30 m × 0.25 mm, 0.50 μm film thickness, Agilent Technologies, USA) was used for the initial separation. Helium was the carrier at a flow rate of 1.2 mL·min^−1^. The initial oven temperature was 50 °C, held for 1 min, and then increased to 220 °C at a rate of 5 °C per min and held for 5 min. The MS transfer line temperature was 280 °C. The mass spectrometric conditions were set as follows: electron ionization (EI) energy at 70 eV and ion source temperature at 230 °C. High-purity nitrogen gas was used as collision-activated dissociation gas. Both 2-AP and 2,6-DMP were quantified using the optimized MRM parameters, as summarized in Table S1. The data were recorded and processed using the MassHunter software (version 07.06, Agilent Technologies, Santa Clara, CA, USA).

### 2.6. Profiling, Identification, and Relative Quantification of Volatile Compounds Using HS-SPME-GC-MS

The profiling, identification, and relative quantification of volatile compounds were carried out using the HS-SPME-GC–MS system (Agilent Technologies, USA). The separation was achieved with a DB-WAX column (30 m × 0.25 mm, 0.50 μm film thickness, Agilent Technologies, USA) using helium as a carrier gas at a constant flow rate of 1.2 mL·min^−1^. All other gas chromatography conditions were the same as those in Section 2.5. The mass spectrometer was operated at EI mode with ionization energy of 70 eV and a source temperature of 230 °C. The full-scan mode (MS1) was used for the volatile profiling with a mass range from m/z 50 to 600. The scan time was 250 ms. The data were recorded and processed using MassHunter 4.5 software (Agilent Technologies, USA).

The volatile compounds were identified by matching mass spectra with mass spectral libraries such as NIST, Wiley, and Fiehn GC–MS libraries, and comparing them with retention index (RI) values reported in the literature. The identification of some compounds was also confirmed using authentic standards such as 2-AP, indole, naphthalene, and 1-hexanol. The RI values were obtained from the analysis of a mixture of n-alkane (C7–C40) under similar conditions [11,12]. The relative concentration of identified volatile compounds were expressed relating to the internal standard, 2,6-DMP [13].

### 2.7. Data Processing and Statistical Analysis

The raw GC-MS data Agilent files (D), acquired in scan mode, were converted to mzML format using the MSConvert (ProteoWizard Palo Alto, CA, USA) software. Peak detection, isotopic peak grouping, peak list alignment, and gap-filling were conducted using MZmine 2.5 [14]. The processed data were exported to a csv. file for further analysis. Data were median normalized, auto-scaled and subjected to multivariate analysis using the MetaboAnalyst 5.0 [15]. Statistical significance (*p* < 0.5) was assessed using the SPSS software (v. 23, BM SPSS Statistics, IBM Corp., Chicago, IL, USA). The results were expressed as a mean ± standard error (SE) of three to four replicates. MS Excel was used for regression analysis and data visualization.

## 3. Results and Discussion

### 3.1. Optimization of HS-SPME Parameters

Milled rice, of the variety Pusa Basmati-1121, was used for the optimization of HS-SPME conditions. Initially, the extraction performance of volatile compounds in rice sample was tested using three different SPME fibers, such as CAR/PDMS, PDMS/DVB, and 50/30 µm DVB/CAR/PDMS, by comparing the abundance of volatile compounds in the respective total ion chromatograms (Figure S2). This study showed that 50/30 µm DVB/CAR/PDMS fiber extracted the maximum number of volatile compounds, as found in the previous studies [9,14]. In subsequent steps, using 50/30 µm DVB/CAR/PDMS fiber, the optimal incubation temperature and extraction time were estimated to extract the maximum amount of 2-AP from the raw rice sample at three different sample weights. Figure S3 shows the results of the optimization of HS-SPME conditions for the extraction of 2-AP at different incubation temperatures, extraction times, and sample weights. The highest recovery of 2-AP at the shorter extraction time and minimal incubation temperature was the main criteria for determining the optimal extraction conditions. In this study, all the studied parameters, such as incubation temperature, extraction time, and sample weight, were found to have a considerable influence on the extraction yield of 2-AP. An increase in the 2-AP content was observed with an increase in incubation temperature and extraction time up to 60 °C and 15 min, respectively. Higher incubation temperature and extraction time above 60 °C and 15 min, respectively, led to either no change or a decrease in the 2-AP level. Similarly, at the 1.0 g sample weight, the extraction yield of 2-AP was maximum. These findings, altogether, showed that one gram of sample incubated at 60 °C for 15 min was the optimal HS-SPME condition to extract the amount of 2-AP from the raw rice sample. Previous studies also have shown 60 °C as the optimal incubation temperature for the extraction of 2-AP and other volatiles [16,17]. In several other studies, 2-AP from rice samples was extracted at a higher incubation temperature, ≤80 °C [6,18,19]. However, as seen in this study, the extraction temperature over 75 °C was not found to be suitable to obtain an optimal yield of 2-AP; this is possibly because of a loss of volatiles, including 2-AP, occurring at high extraction temperature may be due to the failure of the vial cap septum [9,19]. As per the literature, an equilibration time of 10–15 min is generally sufficient to extract the majority of volatile compounds. Akin to the findings of this study, an extraction time of 15 min was found to be optimal to achieve a maximum yield of 2-AP from rice [20].

In previous studies, rice samples were cooked in the headspace vial by adding water [8,20]. However, the amount of water added in proportion to the weight of the rice sample was found to be crucial to obtain a maximum yield of 2-AP from cooked rice by HS-SPME [20]. Considering this, the amount of water required for the optimal extraction of 2-AP from a cooked rice sample was optimized at 60 °C for 15 min incubation in a subsequent study. Compared to parts of water (mL) to rice (g) ratios of 0.5:1.0, 1.0:1.0, and 2.0:1.0, a smaller amount of water (0.250 mL) added to the rice (1.0 g) considerably increased the amount of 2-AP (Figure S3b). The addition of water over 0.25 mL into the one-gram rice sample containing vial was found to remarkably reduce the extraction yield of 2-AP. Taken together, the incubation of a 20 mL vial containing one gram of rice grain sample and 0.25 mL of water at 60 °C for 15 min was the optimal HS-SPME experimental condition for the extraction of 2-AP from cooked rice.

### 3.2. 2-AP Contents in Raw and Cooked Rice

**Figure 1:**
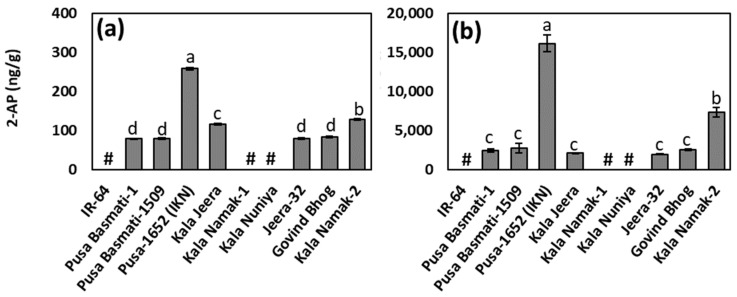
(**a**) 2-acetyl-1-pyrroline (2-AP) content in raw, and (**b**) cooked rice samples of ten different traditional and improved varieties grown in the wet-season of 2020. The 2-AP content below the level of detection is indicated as hash (#). Different letters indicate significant differences at *p* < 0.05, as determined by Tukey’s test. Pusa-1652 (Improved Kala Namak) is abbreviated as Pusa-1652 (IKN).

The results of 2-AP contents in raw and cooked rice of ten different traditional and improved varieties grown in the wet season of 2020 are shown in Figure 1. In raw rice samples, 2-AP content was the highest in Pusa 1652 (Improved Kala Namak), followed by Kala Namak-2 and Kala Jeera (Figure 1a). The 2-AP level in the Pusa 1652 (Improved Kala Namak) raw rice sample was almost double of that in Kala Namak-2. No significant differences were found in the 2-AP levels of Pusa Basmati-1, Pusa Basmati-1509, Jeera-32, and Govind Bhog (Figure 1a). The rice samples of Kala Namak-1 and Kala Nuniya had 2-AP levels below the level of detection, similar to that observed in the non-aromatic rice variety, IR-64. Considering that Kala Namak is well-known aromatic rice, the aromatic property of the Kala Namak 1 sample similar to a non-aromatic rice with respect to 2-AP raises doubt on the authenticity of this sample used in our study.

Figure 1b shows the 2-AP levels in the cooked rice samples. The cooked rice samples exhibited approximately 20-to-60-fold increase in the amount of 2-AP compared to the corresponding raw rice samples, demonstrating that 2-AP may be significantly increased during cooking. Akin to the trend observed in raw rice samples, the cooked sample of Pusa 1652 (Improved Kala Namak) had the highest amount of 2-AP compared to all other cooked samples. For the rest of the samples also, the pattern of 2-AP content was almost similar, as seen in their raw samples. The observed fold increase in the 2-AP content in cooked rice of Pusa 1652 (Improved Kala Namak) (63-fold) and Kala Namak-2 (57-fold) rice samples was higher than the Pusa Basmati-1509 (35-fold), Pusa Basmati-1 (31-fold), Govind Bhog (31-fold), Jeera-32 (25-fold), and Kala Jeera (18-fold). The Pusa 1652 (Improved Kala Namak) line was derived from a cross of two aromatic donors, traditional Kalanamak and Pusa 1176, and further backcrossed to Kalanamak. Pusa 1176 was derived from Bindli Mutant 68 (BM68) X ARC line. The BM 68 is a mutant of Bindli, a traditional tall short grain aromatic rice line. For non-aromatic rice, IR 64 and the two mild-aromatic 2AP rice samples, Kala Nuniya and Kalanamak 1, the fold increase in the 2-AP content was not detected.

The 2-AP contents in raw and cooked rice samples of the above varieties were also estimated in the stored material that were grown and harvested in 2019 (Figure S4). The observed 2-AP levels in the raw and cooked rice samples of 2019 were lower than those harvested in 2020. Interestingly, the 2-AP levels in the raw rice samples of all varieties studied were below the level of detection (Figure S4a). However, statistically significant variations in 2-AP levels were found in the cooked rice (Figure S4b). The cooked sample of Pusa 1652 (Improved Kala Namak) from the year 2019 also had the highest 2-AP content, followed by Kala Namak-2 and Kala Jeera. The observed fold increase in 2-AP content due to cooking of 2019 samples was also lower than the increase observed in freshly harvested rice samples of 2020. Similar to 2020 harvested samples, the non-aromatic and the mild-aromatic genotypes harvested in 2019 did not show fold increase in the 2-AP content. For aromatic genotypes, the observed fold increase in the 2-AP content was in the range of 10.8–29.9. Collectively, these findings indicate that significant loss occurs in 2-AP content during storage, as reported previously [9,21]. Additionally, compared to raw rice, the analysis of cooked rice samples could be crucial to understanding the aromatic nature of rice.

### 3.3. Untargeted Analysis of Volatile Profiles of Raw and Cooked Rice Samples

**Figure 2:**
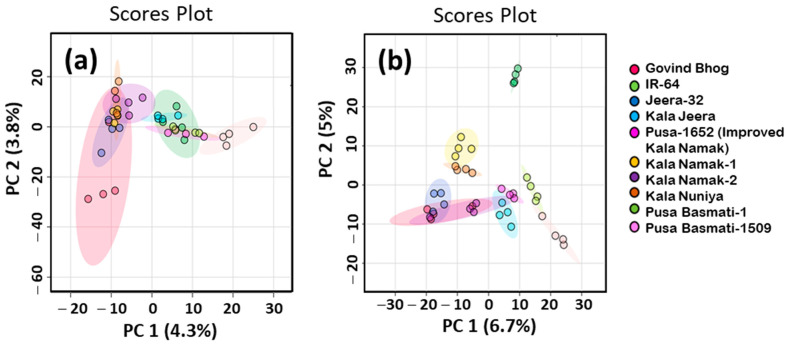
Principal component analysis (PCA) score plots of volatile compounds in (**a**) raw and (**b**) cooked rice samples of ten different traditional and improved varieties.

To explore the proximity among volatile profile datasets of raw and cooked rice samples of varieties studied, untargeted metabolomics analyses were performed. The results of principal component analysis (PCA) of the volatile profile dataset of raw and cooked rice samples are shown in Figure 2. PCA is the initial step in multivariate data analysis, which is routinely performed to understand the diversity pattern among the dataset [22]. Taking into consideration the principal components PC1 and PC2, only 8.1% of phenotypic variance is accounted for volatile profiles in the raw rice samples (Figure 2a). However, cooked rice samples of some varieties studied showed distinct grouping according to their aromatic and non-aromatic nature (Figure 2b). However, the observed total percentage of variance explained by PC1 and PC2 was only 11.7%, which indicates the high number of independent sources of variability in the matrix. Cooked rice samples of IR-64 formed a well-separated cluster from other varieties in the PCA scores plot. The mild-aromatic traditional varieties, Kala Namak-1 and Kala Nuniya, were clustered together and considerably discriminated from non-aromatic IR-64 and aromatic varieties. In this PCA plot, among aromatic varieties, Pusa Basmati-1 and Pusa Basmati-1509 had a separate cluster, which was detached from the other traditional aromatic varieties. Interestingly, traditional aromatic landraces were grouped together, which demonstrates close similarity among their volatile profiles. Altogether, these results indicated that the volatile profiles of aromatic and non-aromatic rice varieties were more distinguishable in cooked rice than their raw samples.

### 3.4. Volatile Profiles of Raw and Cooked Rice Samples

The identified volatile compounds in raw and cooked rice samples are tabulated in Table 1 and Table 2. Altogether, 18 and 22 volatiles were identified in the raw and cooked rice samples of varieties studied, respectively. The identification of these compounds was carried out based on mass spectra, retention index (RI) values, and authentic standards (Table 1 and Table 2). The relative concentration of the identified volatile compounds in raw and cooked rice samples among studied varieties are also depicted in Table 1 and Table 2. The volatile compounds, 1-hexanol, and ethyl 3-hydroxybutyrate, were common in the raw rice samples of all varieties studied. The aliphatic aldehydes and alcohols, such as 1-hexanol and nonanal, are lipid oxidation products and were reported to be responsible for aroma intensities of aromatic and non-aromatic rice [23,24]. The content 2-ethyl-1-hexanol was highest in the raw rice samples of most of the varieties, except IR-64 and Govind Bhog. Interestingly, 2-ethyl-1-hexanol was not detected in the cooked rice samples, which showed that it can be lost in the cooking process and can be a volatile marker compound for freshly milled rice samples. The 2-ethyl-1-hexanol is the contributor for citrus, fresh floral, oily, and sweet aromas, and is identified as a discriminating compound for Basmati brand B, and a maker for rice aging in certain varieties in previous studies [25,26].

The raw rice samples of Pusa-1652 (Improved Kala Namak) had almost all identified volatile compounds in detectable amounts, except ethyl hexanoate and 1,3-Butanediol. The raw rice of Pusa-1652 (Improved Kala Namak) depicted significantly higher 2-AP, phenylethyl alcohol, and indole contents compared with raw rice samples of other varieties. The contents of nonanoic acid, ethyl ester, 2,3-butanediol, 1-octanol, tetradecanoic acid, ethyl ester, and ethyl 9-hexadecenoate were significantly higher in the raw rice samples of Pusa Basmati-1509. The raw rice samples of the traditional variety, Kala Namak-2, had the highest content of 2-AP after Pusa-1652 (improved Kala Namak). Both Pusa-1652 (Improved Kala Namak) and Kala Namak-2 had a significantly higher amount of 1-octen-3-ol. The 1-octen-3-ol is an off-flavor-causing component responsible for green, mushroom, earthy, and oily aromas, which was reduced or lost after cooking in these varieties [27].

The content of certain volatile compounds was found to be increased after cooking, but this increase was rice-genotype-specific (Table 2). Interestingly, the volatile compounds such as ethyl butyrate, ethyl 3-methylbutanoate, 2-undecanone, ethyl benzoate, ethyl benzeneacetate, 2-methylnaphthalene, and 1-methylnaphthalene were characteristically detected in the cooked rice samples. This finding demonstrated that these volatile compounds were either released or formed during cooking. Both ethyl butyrate and ethyl 3-methylbutanoate were detected together in Pusa-1652 (Improved Kala Namak), and Kala Namak-2 cooked rice samples. The highest amount of 2-AP was found in Pusa-1652 (Improved Kala Namak), followed by Kala Namak-2, Govind Bhog, and Kala Jeera. Cooked rice of Pusa Basmati-1, Pusa Basmati-1509, and Jeera-32 had an almost similar amount of 2-AP. Nonanal content was highest in the cooked rice samples of Pusa Basmati-1509. Notably, 1-octanol was observed in cooked rice samples of all varieties, except Pusa-1652 (Improved Kala Namak). An increase in indole content was observed in the cooked rice samples of IR-64 and Kala Namak-2. Conversely, it decreased to an undetectable level after cooking in Pusa-1652 (Improved Kala Namak).

Although the perceived aroma of particular rice after cooking is the outcome of the combined effects of all the aromatic volatiles present, the odor predominance may be skewed towards the effect of the most dominant volatile compounds present. Amongst the rice varieties studied here, 2-AP was the most dominant compound found only in Pusa 1652 (Improved Kala Namak) and Kala Namak 2. Given the unique potential of Pusa 1652 (Improved Kala Namak), as a novel source of enhanced aroma due to the detection of very high levels of 2AP and other volatile signatures found in this improved Kalanamak variety, the genetics of improved aroma signatures of Pusa 1652 need to be deciphered in future studies. The second dominant compound in these two rice samples was ethyl hexanoate and 1-hexanol, characteristic for a fruity apple and green herbaceous woody odor, respectively. Four of the rice varieties studied, Pusa Basmati-1, Kala Jeera, Kala Namak-1, and Kala Nuniya, had 1-hexanol as the most dominant volatile compound, whereas two others, Pusa Basmati 1509 and Govind Bhog, had ethyl hexanoate as the most dominant volatile compound. The findings of this study collectively confirmed that several of the aromatic traits of traditional varieties exhibited a Basmati-like aroma which was equivalent to or superior to Basmati rice. Jeera 32 is the only rice variety studied that stood unique, with ethyl benzoate present as the most dominant volatile compound. These results point out the importance of deploying the targeted and untargeted metabolomics techniques to identify the important donor lines with very high level of 2AP and unique aroma signatures to be deployed for future breeding to diversify aroma and to maintain the stability of aroma. It would be interesting to see if a descriptive sensory panel-based study will categorize these rice varieties in a manner similar to the differentiation based on the effect of dominant volatile compounds present after cooking.

**Figure 3:**
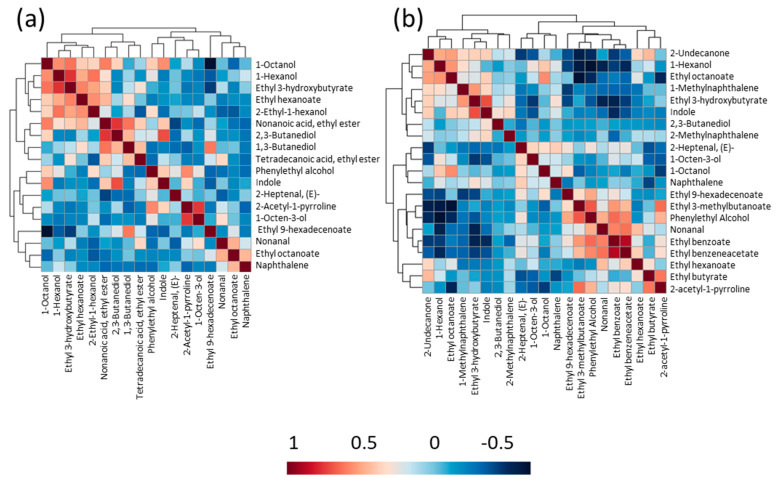
Heatmap of correlation matrix of the volatile compounds in (**a**) raw rice and (**b**) cooked rice of ten different traditional and improved varieties. Red-colored squares indicate positive correlations and blue-colored squares denote negative correlation.

The heatmap of the correlation matrix was plotted to understand the relationship between volatile compounds in raw and cooked rice samples (Figure 3). In the correlation heatmap, red-colored squares indicate positive corrections, and compounds in blue-colored squares showed negative or non-significant correlations. A strong positive correlation was found in raw rice samples between 1-hexanol and ethyl 3-hydroxybutyrate, nonanoic acid, ethyl ester, and 2,3-butanediol, 2-AP and 1-octen-3-ol (Figure 3a). These findings showed that with 2-AP, 1-octen-3-ol might contribute to the perceived scent of raw aromatic rice samples, as reported in the previous study [28]. Conversely, a strong negative correlation was observed among tetradecanoic acid, ethyl ester, and phenylethyl alcohol.

A strong positive correlation was observed between ethyl benzoate and ethyl benzeneacetate in cooked rice, which indicates they may be formed together during the cooking process. 2-AP showed moderate to high correlations with ethyl benzoate, ethyl benzeneacetate, ethyl butyrate, ethyl 3-methylbutanoate and phenylethyl alchohol, ethyl 3-hydroxybutyrate, and indole, which demonstrates the role of these volatile compounds to the perceived aroma of cooked aromatic rice samples (Figure 3b). The observed moderate to the high correlation of 2-AP with ethyl benzoate, benzene acetic acid, ethyl ester, ethyl butyrate, ethyl 3-methylbutanoate, and phenylethyl alchohol, ethyl 3-hydroxybutyrate, and indole in cooked rice suggests the role of these compounds in the overall perceived characteristic Basmati-like aroma in cooked rice.

## 4. Conclusions

The present finding highlights important inferences, such as the fact that monitoring cooked rice volatiles has specific advantages over the traditional method of raw rice profiling. The present study demonstrated that Kala Namak varieties such as Pusa-1652 (Improved Kala Namak) and Kala Namak-2 were superior in the 2-AP content than Basmati varieties. Moreover, the study revealed that the 2-AP content considerably increased after cooking, which might be attractive to consumers. As 2-AP is shown to have a reduced effect with storage, for aromatic segments, it is important to use either the fresh harvests or store the samples at refrigerated conditions to maintain a higher aroma. This study showed that the cooked rice was the best matrix to distinguish volatile compound profiles of aromatic and non-aromatic rice varieties. In addition, untargeted profiling combined with targeted profiling of 2AP has identified several unique fruity odor targets in non-basmati rice varieties. Of these, ethyl butyrate (fruity green), ethyl 3-methylbutanoate (fruity sweet apple), ethyl benzoate (sweet fruity), 2-methylnaphthalene (sweet floral), and unknown odor contributing volatiles 1-methylnaphthalene, ethyl benzeneacetate were characteristically detected at higher levels in several aromatic varieties in the cooked rice samples. The deployment of volatilomics technologies to quantify 2-AP content reliably and the profiling of other aromatic volatile compounds through an untargeted approach further helped to identify unique rice landraces that could potentially be commercialized under a geographical indicator (GI) tag and to provide genetically diverse donor parents for crop improvement programs. Moreover, the dominant volatile compounds detected in each of the rice varieties could potentially serve as biochemical markers that may assist in detecting adulteration and determining the authenticity of the genotype. The study also sheds light into potentially diversifying the aroma signatures in pre-breeding and breeding for targeting it as premium quality rice with superior and distinct aroma.

## Figures and Tables

**Table 1:** Identified major volatile compounds and their relative levels in raw rice samples of ten traditional and improved varieties.

Compound	RI_cal_	^a^ RI_rep_	Identi-fication Details	^b^ Odor Description	Concentration in ng/g									
					IR-64	Pusa Basmati-1	Pusa Basmati-1509	Pusa-1652 (Improved Kala Namak)	Kala Jeera	Kala Namak-1	Kala Nuniya	Jeera-32	Govind Bhog	Kala Namak-2
Ethyl hexanoate	1242	1240	MS, RI	Fruity, apple peel	n.d.	n.d.	1.2 ± 0.1a	n.d.	n.d.	n.d.	n.d.	1.1 ± 0.0a	n.d.	n.d.
2-Heptenal, (E)-	1338	1338	MS, RI	Fruity, green, fatty	n.d.	2.4 ± 0.5a	n.d.	1.7 ± 0.1a	1.8 ± 0.1a	n.d.	n.d.	n.d.	n.d.	n.d.
2-Acetyl-1-pyrroline	1354	1343	MS, ST	Popcorn, toasted grain, nuty	n.d.	n.d.	n.d.	1.8 ± 0.2a	0.5 ± 0.0c	n.d.	n.d.	n.d.	n.d.	0.9 ± 0.1b
1-Hexanol	1361	1363	MS, RI, ST	Green, herbaceous, woody, sweet	1.4 ± 0.1d	2.6 ± 0.1abcd	1.7 ± 0.6cd	2.9 ± 0.0abc	2.3 ± 0.4abcd	1.7 ± 0.0cd	3.2 ± 0.3ab	2.5 ± 0.0abcd	3.5 ± 0.1a	2.0 ± 0.2bcd
Nonanal	1401	1396	MS, RI	Green, fatty, citrus	1.3 ± 0.1c	2.5 ± 0.2ab	2.4 ± 0.4abc	2.6 ± 0.1ab	n.d.	1.2 ± 0.0c	n.d.	n.d.	1.5 ± 0.2bc	2.8 ± 0.6a
Ethyl octanoate	1444	1444	MS, RI	Fruity, fatty, brandy	n.d.	2.2 ± 0.2a	1.9 ± 0.3a	1.6 ± 0.1ab	n.d.	n.d.	n.d.	n.d.	1.2 ± 0.1b	1.3 ± 0.1b
1-Octen-3-ol	1455	1460	MS, RI	Green, mushroom, earthy, oily	n.d.	n.d.	1.6 ± 0.2b	2.8 ± 0.2a	1.8 ± 0.3b	1.2 ± 0.0b	n.d.	n.d.	n.d.	3.1 ± 0.3a
2-Ethyl-1-hexanol	1494	1490	MS, RI	Citrus, fresh floral, oily, sweet	n.d.	25.9 ± 3.3a	26.2 ± 0.6a	28.0 ± 0.4a	24.1 ± 3.5a	32.3 ± 0.4a	29.3 ± 1.8a	28.2 ± 4.0a	n.d.	30.5 ± 0.9a
Ethyl 3-hydroxybutyrate	1529	1524	MS, RI	Green, fruity, waxy, apple skin	2.7 ± 0.2a	2.7 ± 0.2a	2.3 ± 0.1a	2.0 ± 0.2a	2.7 ± 0.1a	2.6 ± 0.2a	2.2 ± 0.1a	2.6 ± 0.1a	2.4 ± 0.1a	2.0 ± 0.0a
Nonanoic acid, ethyl ester	1542	1541	MS, RI	Waxy, fruity, nutty, wine-note	n.d.	n.d.	2.2 ± 0.1b	2.8 ± 0.2a	n.d.	n.d.	n.d.	n.d.	n.d.	n.d.
2,3-Butanediol	1548	1545	MS, RI	Creamy, fruity, buttery	n.d.	n.d.	3.9 ± 0.7b	7.6 ± 0.0a	n.d.	n.d.	n.d.	n.d.	n.d.	n.d.
1-Octanol	1564	1565	MS, RI	Waxy, green citrus	n.d.	n.d.	n.d.	1.5 ± 0.2a	n.d.	n.d.	n.d.	n.d.	n.d.	n.d.
1,3-Butanediol	1582	1578	MS, RI	-	n.d.	n.d.	7.6 ± 2.2a	n.d.	n.d.	n.d.	n.d.	n.d.	n.d.	n.d.
Naphthalene	1764	1755	MS, ST	Pungent, tarry	n.d.	2.4 ± 0.5	n.d.	2.3 ± 0.3a	0.9 ± 0.5ab	1.3 ± 0.0bc	n.d.	n.d.	2.0 ± 0.2ab	n.d.
Phenylethyl alcohol	1929	1924	MS, RI	Floral, sweet, rosey, honey	n.d.	1.3 ± 0.1c	n.d.	6.7 ± 0.1a	1.8 ± 0.1b	n.d.	1.3 ± 0.0c	n.d.	n.d.	1.7 ± 0.1b
Tetradecanoic acid, ethyl ester	2060	2067	MS	Waxy, sweet	3.2 ± 0.2b	2.6 ± 0.3bc	8.1 ± 0.3a	1.7 ± 0.1d	1.9 ± 0.0cd	n.d.	n.d.	n.d.	n.d.	n.d.
Ethyl 9-hexadecenoate	2267	2267	MS, RI	-	n.d.	25.9 ± 2.5b	65.8 ± 0.7a	7.8 ± 0.4cd	11.1 ± 0.5c	2.4 ± 0.3fg	2.0 ± 0.1fg	2.9 ± 0.4efg	4.4 ± 0.2def	6.7 ± 0.2de
Indole	2475	2478	MS, RI, ST	Animal, floral, mothball	3.6 ± 0.2b	n.d.	n.d.	60.9 ± 0.4a	n.d.	n.d.	n.d.	n.d.	n.d.	n.d.

RI_cal_: calculated retention index, MS: mass spectrum, ST: authentic standard, n.d.: not detected. ^a^ RI_rep_: reported RI value obtained in 21–31 January 2021 from https://webbook.nist.gov/. ^b^ Odor description retrieved 10 February 2021 from http://www.thegoodscentscompany.com or https://www.flavornet.org/. Different letters in the same row indicated statistically significant differences.

**Table 2:** Identified major volatile compounds and their relative levels in cooked rice samples of ten traditional and improved varieties.

Compound	RI_cal_	^a^ RI_rep_	Identi-Fication Details	^b^ Odor Description	Concentration in ng/g									
					IR-64	Pusa Basmati-1	Pusa Basmati-1509	Pusa-1652 (Improved Kala Namak)	Kala Jeera	Kala Namak-1	Kala Nuniya	Jeera-32	Govind Bhog	Kala Namak-2
Ethyl butyrate	1046	1041	MS, RI	Fruity, green, apple, fatty	n.d.	5.1 ± 1.1abc	8.1 ± 1.4a	7.0 ± 0.9ab	3.7 ± 0.5b	n.d.	3.4 ± 0.5cd	n.d.	n.d.	4.1 ± 0.9bc
Ethyl 3-methylbutanoate	1077	1078	MS, RI	Fruity, sweet apple, pineapple	n.d.	n.d.	n.d.	8.1 ± 0.4a	n.d.	n.d.	n.d	5.7 ± 0.8ab	6.5 ± 1.7ab	3.9 ± 0.6b
Ethyl hexanoate	1242	1240	MS, RI	Fruity, apple peel	11.1 ± 0.2def	33.8 ± 1.4ab	44.5 ± 1.9a	19.8 ± 1.9cd	22.7 ± 3.4bc	8.2 ± 0.4ef	12.9 ± 3.0 cde	15.0 ± 0.8cde	33.5 ± 4.6ab	n.d.
2-Heptenal, (E)-	1338	1338	MS, RI	Fruity, green, fatty	n.d.	5.6 ± 0.5ab	4.1 ± 0.4bc	n.d.	n.d.	5.2 ± 1.2abc	4.0 ± 0.8bc	6.6 ± 2.8ab	10.0 ± 1.5a	5.2 ± 0.1abc
2-Acetyl-1-pyrroline	1354	1343	MS, ST	Popcorn, toasted grain, nuty	n.d.	15.6 ± 1.2d	16.2 ± 0.8d	70.4 ± 2.0a	21.1 ± 1.3c	n.d.	n.d.	14.7 ± 1.3d	20.3 ± 1.6c	25.0 ± 0.8b
1-Hexanol	1361	1363	MS, RI, ST	Green, herbaceous, woody, sweet	7.7 ± 0.5cd	37.7 ± 1.7ab	9.2 ± 0.2cd	4.8 ± 0.4cd	35.9 ± 1.8ab	28.4 ± 1.8abc	45.1 ± 15.9a	n.d.	n.d.	18.2 ± 2.7bcd
Nonanal	1401	1396	MS, RI	Green, fatty, citrus	n.d.	n.d.	34.8 ± 0.7a	10.6 ± 0.4b	n.d.	n.d.	n.d.	14.2 ± 1.7b	10.2 ± 2.5b	n.d.
Ethyl octanoate	1444	1444	MS, RI	Fruity, fatty, brandy	8.9 ± 1.4ab	11.1 ± 0.8ab	10.9 ± 1.1ab	n.d.	12.4 ± 0.3a	6.4 ± 0.6b	10.9 ± 0.7ab	7.2 ± 2.1ab	8.5 ± 1.7ab	9.5 ± 0.4ab
1-Octen-3-ol	1455	1460	MS, RI	Green, mushroom, earthy, oily	n.d.	5.7 ± 0.3ab	5.4 ± 0.8ab	n.d.	5.8 ± 0.8ab	7.6 ± 2.7a	5.7 ± 0.8ab	6.3 ± 0.6ab	9.7 ± 0.8a	2.6 ± 0.4bc
Ethyl 3-hydroxybutyrate	1529	1524	MS, RI	Green, fruity, waxy, apple skin	9.1 ± 0.6a	6.2 ± 0.8ab	n.d.	6.8 ± 0.5ab	5.7 ± 1.2ab	4.1 ± 1.4b	4.6 ± 0.1b	3.8 ± 0.6b	3.5 ± 0.3bc	5.6 ± 0.7ab
2,3-Butanediol	1548	1545	MS, RI	Creamy, fruity, buttery	5.0 ± 0.0a	5.7 ± 0.4a	6.6 ± 1.1a	4.9 ± 0.6a	3.1 ± 0.6ab	4.7 ± 1.7a	n.d.	6.0 ± 1.7a	4.5 ± 0.6a	5.0 ± 0.4a
1-Octanol	1564	1565	MS, RI	Waxy, green citrus	5.0 ± 0.7bc	9.6 ± 1.7ab	11.1 ± 0.5ab	n.d.	9.2 ± 0.7ab	7.8 ± 1.7ab	5.5 ± 0.5abc	8.3 ± 0.5ab	9.0 ± 1.2ab	12.7 ± 4.0a
2-Undecanone	1606	1596	MS	Waxy, fruity creamy, fatty, floral	7.1 ± 1.3abc	10.3 ± 0.4a	10.3 ± 1.4a	5.2 ± 0.4bc	7.9 ± 1.1ab	2.9 ± 1.7cd	5.0 ± 0.8ab	n.d.	n.d.	4.7 ± 0.6bc
Ethyl benzoate	1682	1677	MS, RI	Sweet, fruity, wintergreen, medicinal,	n.d.	5.6 ± 1.2de	16.1 ± 3.1ab	14.7 ± 1.0bc	11.1 ± 0.9bcd	4.6 ± 0.5de	8.6 ± 0.9cd	22.2 ± 2.5a	17.5 ± 0.9ab	7.9 ± 0.1cd
Naphthalene	1764	1755	MS, ST	Pungent, tarry	6.6 ± 1.1b	8.8 ± 0.7ab	8.3 ± 0.6ab	8.2 ± 0.6ab	7.1 ± 0.7ab	8.3 ± 0.5ab	7.8 ± 0.8ab	10.2 ± 0.8a	8.6 ± 0.3ab	7.4 ± 1.0ab
Ethyl benzeneacetate	1798	1782	MS	-	n.d.	n.d.	6.2 ± 1.0abc	5.7 ± 0.6abc	4.0 ± 0.3bc	n.d.	3.2 ± 0.2c	6.7 ± 1.4ab	7.6 ± 0.5a	n.d.
2-Methylnaphthalene	1878	1877	MS, RI	Sweet, floral, woody	3.8 ± 0.4bc	n.d.	5.9 ± 0.5a	4.2 ± 0.1b	2.5 ± 0.3cd	3.2 ± 0.8bcd	n.d.	n.d.	4.6 ± 0.2ab	2.2 ± 0.1d
1- Methylnaphthalene	1916		MS	Naphthyl, medicinal	5.8 ± 1.2a	4.9 ± 0.7a	3.8 ± 0.1a	5.0 ± 1.0a	5.4 ± 0.4a	4.1 ± 0.6a	3.2 ± 0.4ab	4.8 ± 0.9a	n.d.	4.9 ± 0.7a
Phenylethyl Alcohol	1929	1924	MS, RI	Floral, sweet, rosey, honey	n.d.	n.d.	4.5 ± 0.2a	5.1 ± 0.3a	n.d.	n.d.	n.d.	5.0 ± 0.7a	4.8 ± 0.3a	5.6 ± 0.8a
Ethyl 9-hexadecenoate	2267	2267	MS, RI	-	n.d.	n.d.	5.3 ± 1.0a	n.d.	n.d.	n.d.	n.d	5.4 ± 0.3a	8.6 ± 2.7a	6.9 ± 1.3a
Indole	2475	2478	MS, RI, ST	Animal, floral, mothball	28.6 ± 7.3b	n.d.	n.d.	n.d.	n.d.	n.d.	n.d.	n.d.	n.d.	13.0 ± 1.7a

RI_cal_: calculated retention index, MS: mass spectrum, ST: authentic standard, n.d.: not detected. ^a^ RI_rep_: reported RI value obtained in 21–31 January 2021 from https://webbook.nist.gov/. ^b^ Odor description retrieved 10 February 2021 from http://www.thegoodscentscompany.com or https://www.flavornet.org/. Different letters in the same row indicated statistically significant differences.

## Data Availability

Data sharing is not applicable to this article.

## Supplementary Materials

The following are available online at https://www.mdpi.com/article/10.3390/foods10081917/s1, Figure S1: images of paddy and milled rice samples of studied traditional and improved varieties, Figure S2: the total ion chromatogram (TLC) of volatile compounds of rice sample, which were extracted using three different SPME fibers, CAR/PDMS, PDMS/DVB, and 50/30 µm DVB/CAR/PDMS, Figure S3: optimization of SPME conditions. (a) Comparative peak area of 2-acetyl-1-pyrroline (2-AP) at different extraction times (5, 15, and 30 min), incubation temperatures (30, 60, and 90 °C) and sample weights (0.5, 1.0, and 1.5 g). (b) Effect of different amounts of water (0.25, 0.50, and 1.00 mL) addition in 1.0 g rice grain on the peak area of 2-AP after extraction at 60 °C for 15 min. Different letters indicate significant differences at *p* < 0.05, as determined by Tukey’s test, Figure S4: (a) 2-acetyl-1-pyrroline (2-AP) content in raw, and (b) cooked rice samples of ten different traditional and improved varieties grown in the wet season of 2019. The 2-AP content below the level of detection is indicated as hash (#). Different letters indicate significant differences At *p* < 0.05, as determined by Tukey’s test. Pusa-1652 (Improved Kala Namak) is abbreviated as Pusa-1652 (IKN), Table S1: optimized multiple reaction monitoring (MRM) mode parameters for 2-acetyl-1-pyrroline (2-AP) and internal standard 2,6-dimethylpyridine (2,6-DMP). (RT: retention time, CE: collision energy).
