# Supplementary material for: Profiling of 2-Acetyl-1-Pyrroline and Other Volatile Compounds in Raw and Cooked Rice of Traditional and Improved Varieties of India
Source: Foods. 2021 Aug 18;10(8):1917. doi: 10.3390/foods10081917 (PMC8392510; doi:10.3390/foods10081917)
Supplement: Supplementary file 1 [file foods-10-01917-s001.zip › foods-1320858-supplementary.pdf]

Supplementary Material:

Profiling of 2-Acetyl-1-Pyrroline and other Volatile Compounds in Raw and Cooked Rice of Traditional and Improved Varieties of India

Deepak Kasote [1], Vivek Kumar Singh [1], Haritha Bollinedi [2], Ashok Kumar Singh [2], Nese Sreenivasulu [3],* and Ahmed Regina[1]

[1] Centre of Excellence in Rice Value Addition (CERVA), International Rice Research Institute (IRRI) - South Asia Regional Centre (ISARC), Varanasi-221106, Uttar Pradesh (U.P.), India; d.kasote@irri.org (D.K.); vivek.singh@irri.org (V.K.); a.regina@irri.org (A.R.)

[2] Division of Genetics, ICAR- Indian Agricultural Research Institute (IARI), New Delhi, India; haritha.agrico@gmail.com (H.B.); aks_gene@yahoo.com (A.S.)

[3] Consumer-driven Grain Quality and Nutrition Research Unit, International Rice Research Institute, Los Baños, Laguna, 4031, Philippines; n.sreenivasulu@irri.org (N.S.)

* Correspondence: n.sreenivasulu@irri.org (N.S.)

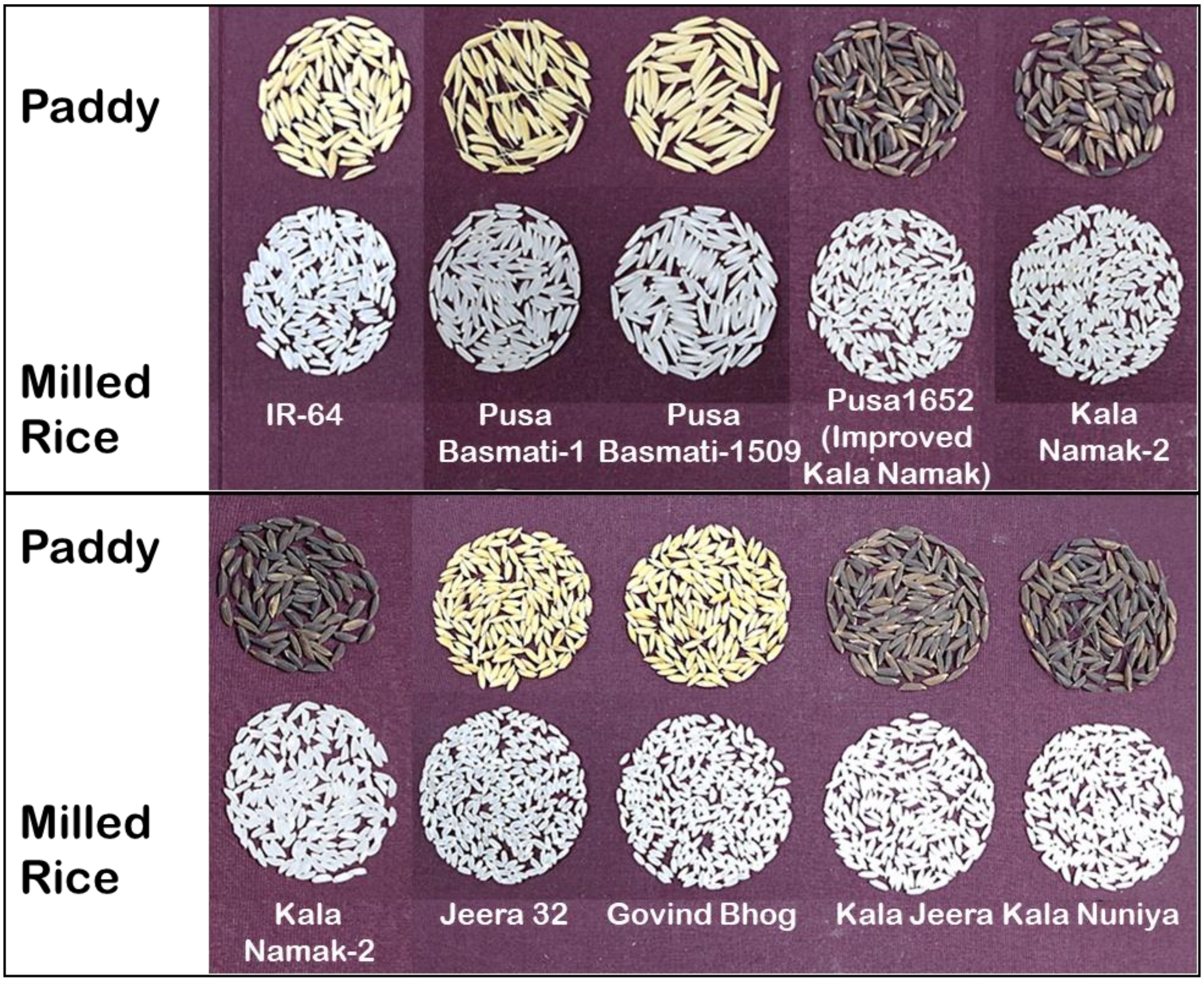

Figure S1. Images of paddy and milled rice samples of studied traditional and improved varieties.

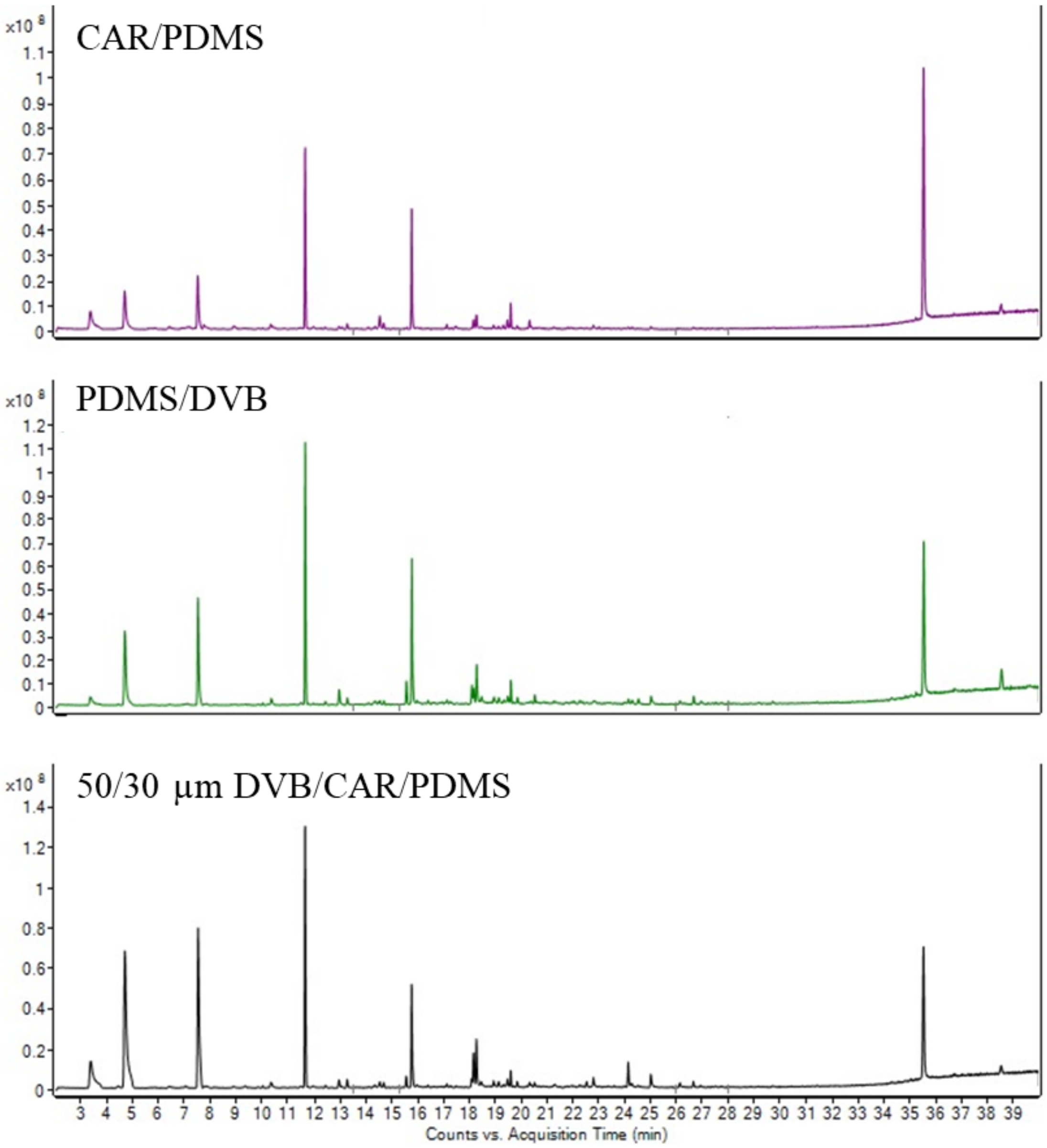

Figure S2: The total ion chromatogram (TLC) of volatile compounds of rice sample, which were extracted using three different SPME fibers, CAR/PDMS, PDMS/DVB, and 50/30 µm DVB/CAR/PDMS.

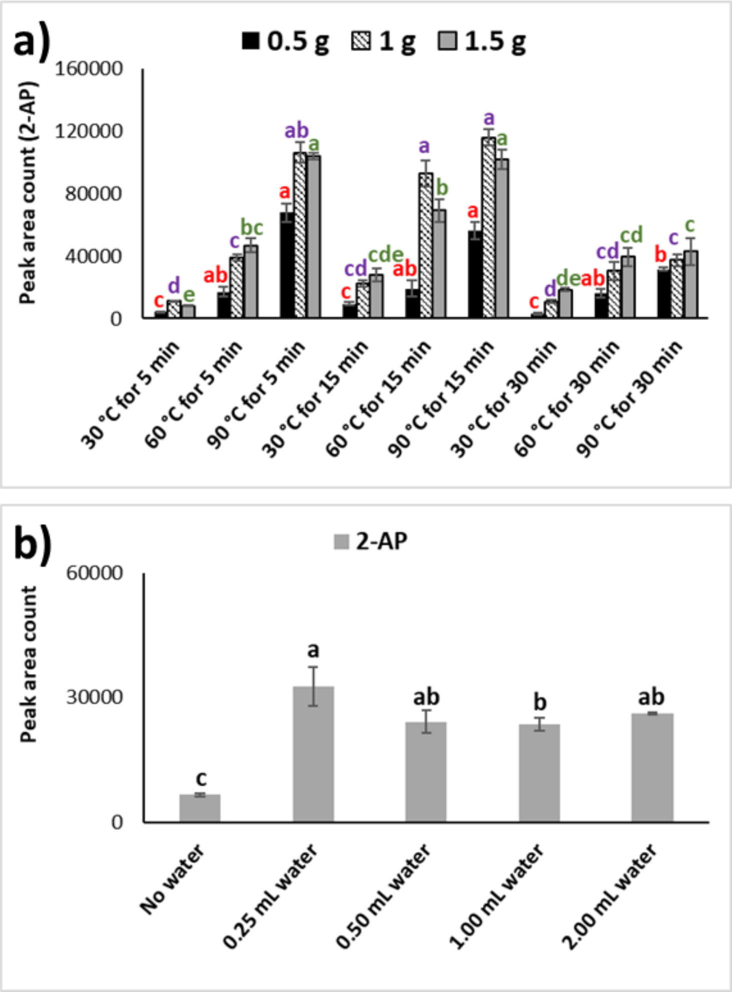

Figure S3. Optimization of SPME conditions. **a)** Comparative peak area of 2-acetyl-1-pyrroline (2-AP) at different extraction times (5, 15, and 30 min), incubation temperatures (30, 60, and 90 ° C) and sample weights (0.5, 1.0, and 1.5 g). **b)** Effect of different amounts of water (0.25, 0.50, and 1.00 mL) addition in 1.0 g rice grain on the peak area of 2-AP after extraction at 60 °C for 15 min. Different letters indicate significant differences at $P < 0.05$, as determined by Tukey's test.

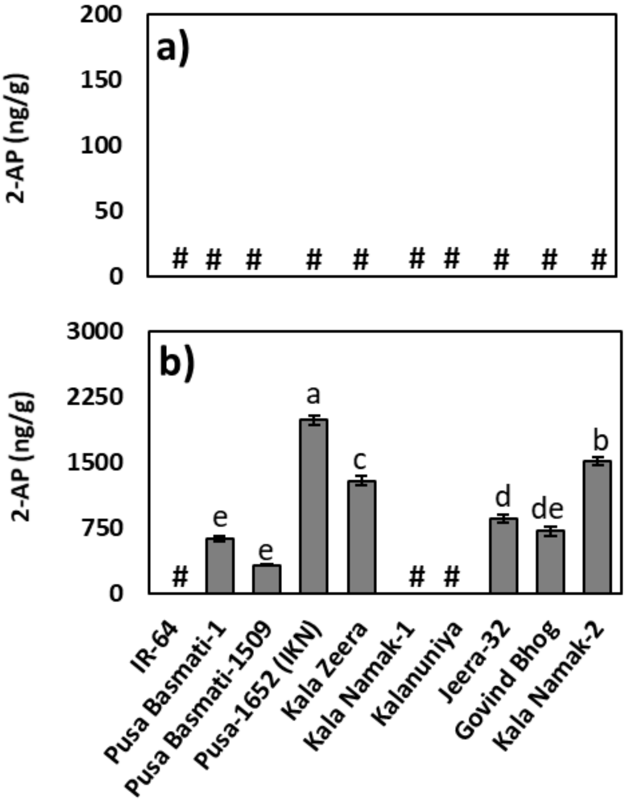

Figure S4: **a)** 2-acetyl-1-pyrroline (2-AP) content in raw, and **b)** cooked rice samples of ten different traditional and improved varieties grown in the wet season of 2019. The 2-AP content below the level of detection is indicated as hash (#). Different letters indicate significant differences at $P < 0.05$, as determined by Tukey's test. Pusa-1652 (Improved Kala Namak) is abbreviated as Pusa-1652 (IKN).

Table S1: Optimized multiple reaction monitoring (MRM) mode parameters for 2-acetyl-1-pyrroline (2-AP) and internal standard 2,6-dimethylpyridine (2,6-DMP). [RT: retention time, CE: collision energy].

Sr. No.	Compound	RT (min)	Transitions	CE (eV)
1.	2,6-DMP	12.062	107.1 → 65.1	25
			107.1 → 92.1	20
			107.1 → 66.1	15
2.	2-AP	14.347	111 → 83	10
			111 → 82	25
